# Inadvertent intrathecal drug injection while treating low back pain: a case report and review of the literature

**DOI:** 10.1186/s13256-023-03754-y

**Published:** 2023-02-17

**Authors:** Alessandro Ferrieri, Donatella Bosco, Ennio Polilli, Raffaella Ciulli, Lina Visocchi, Lucrezia Mincione, Rosa Iacoe, Rosamaria Zocaro, Antonella Frattari

**Affiliations:** 1 Anestesia and Intensive Care Unit, Pescara General Hospital, Via Fonte Romana, 8, 65124 Pescara, PE Italy; 2 Clinical Pathology Unit, Pescara General Hospital, Pescara, Italy

**Keywords:** Undesired intrathecal injections, Chemical meningo-encephalitis, Intrathecal administration

## Abstract

**Background:**

Undesired intrathecal injections represent an important subset of medical errors, albeit rare. Clinical effects depend on the type and concentration of drug(s) injected. Here we report on the case of a healthy woman with persistent low back pain, treated with a paravertebral injection of lidocaine, thiocolchicoside, and l-acetylcarnitine at an orthopedic practice.

**Case report:**

A 42-year-old Caucasian woman, with no relevant past medical history, received a lumbar paravertebral injection of lidocaine, thiocolchicoside, and l-acetylcarnitine for persistent low back pain. Approximately 30 minutes after injection, she experienced quick neurological worsening. Upon arrival at the Emergency Department, she was comatose, with fixed bilateral mydriasis, trismus, and mixed acidosis; seizures ensued in the first hours; slow progressive amelioration was observed by day 6; retrograde amnesia was the only clinical relevant remaining symptom by 6 months.

**Conclusions:**

To our knowledge, this is the first reported case of inadvertent intrathecal thiocolchicoside injection in an adult patient, as well as the first in the neurosurgical literature. Our experience suggests that injection therapy for low back pain should be administered in adequate settings, where possible complications may be promptly treated.

## Introduction and background

The intrathecal space became an important venue for medical interventions in recent years, both in anesthesia and in many other medical specialties [1, 2]. Drugs to be intrathecally administered have to be accurately selected, to ensure that the drugs themselves, their preservatives or adjuncts may not be irritant or harmful to nervous tissues [1, 2]. As a consequence, any medication that may cause neurotoxicity is strictly prohibited from intrathecal use [1, 2].

Undesired intrathecal injections (UII) represent an important subset of medical errors, albeit rare. Clinical effects of UII depend on the type and concentration of drug(s) inadvertently injected, and may range from no adverse reaction to profound morbidity, up to possibly related mortality [2].

Here we report on the case of a healthy woman with persistent low back pain, treated with paravertebral injections of lidocaine, thiocolchicoside, and l-acetylcarnitine in a medical practice, developing rapid neurologic deterioration requiring intensive care admission, with a final diagnosis of chemical meningo-encephalitis.

## Case report

A 42-year-old Caucasian woman, with no relevant past medical history, received a lumbar paravertebral injection of lidocaine, thiocolchicoside, and l-acetylcarnitine at a private orthopedic practice, for persistent low back pain. Approximately 30 minutes after her injection, the patient experienced a rapidly deteriorating neurological condition, with confusion progressing to coma. Upon her arrival at the Emergency Department, the patient was taken in charge by the Medical Emergency Team. At early assessment, she was comatose, with fixed bilateral mydriasis, trismus, and mixed acidosis at hemogasanalysis. Endotracheal intubation and mechanical ventilation were performed, followed by a brain CT scan. During the CT scan, the patient developed seizures, treated with intravenous midazolam. After admission to the Intensive Care Ward, deep sedation with midazolam and remifentanil was started, in addition to levetiracetam and high-dose dexamethasone, as recommended by the local reference guidance center in Rome, which was deliberately consulted.

**Fig. 1:**
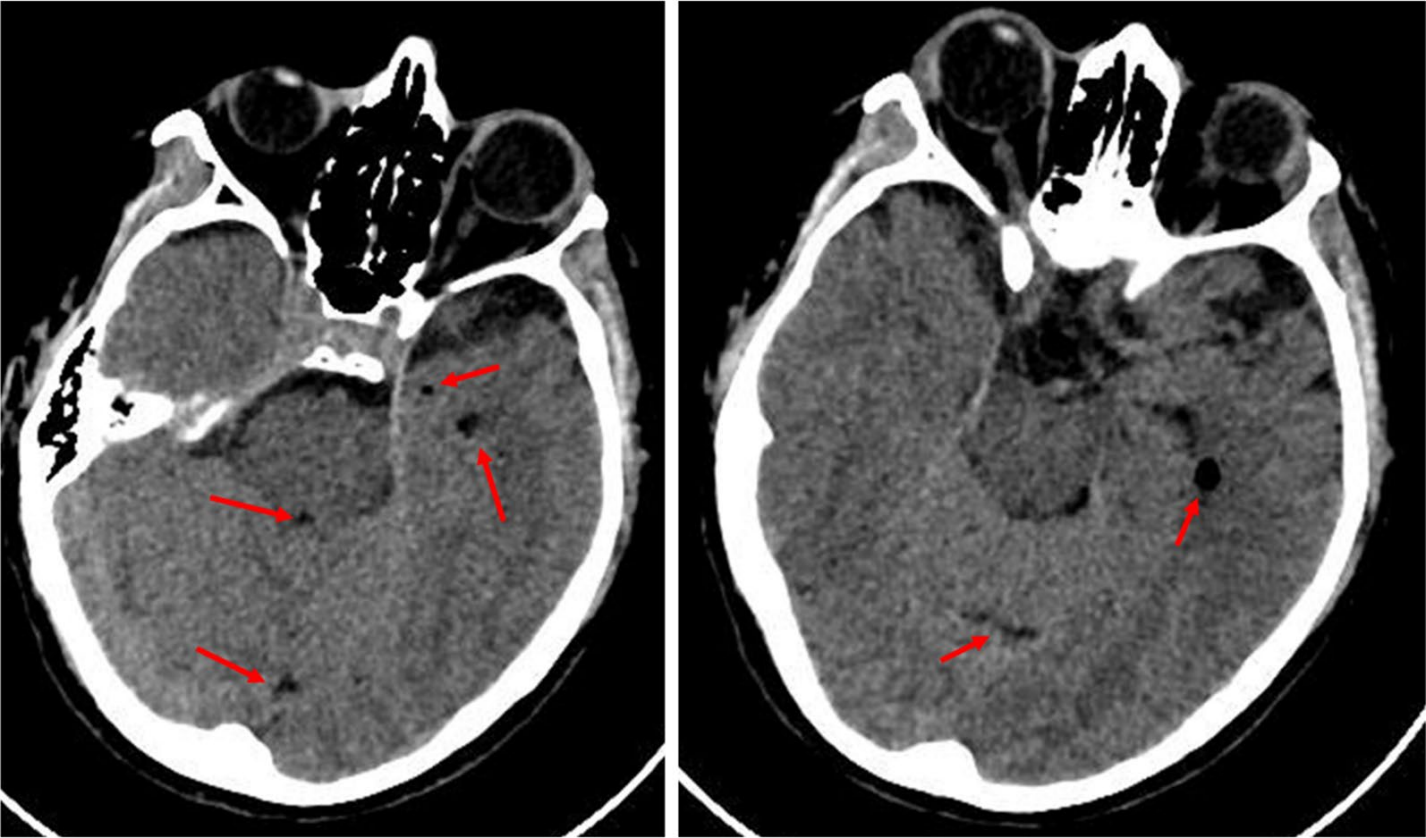
Basal CT scan indicated a thin frontal sub-thecal isodense layer associated with multiple small air bubbles (red arrows)

The initial CT scan indicated a thin frontal sub-thecal isodense layer, associated with multiple small air bubbles in the supratentorial ventricular system, in the perimesencephalic, peripontine, and basal cisterns and in a few periencephalic sulci (Fig. 1). The presence of air bubbles was confirmed by MRI, with no further significant addition. Cerebral spinal fluid (CSF) analysis and cultures were performed. Electroencephalography (EEG) 12 hours after admission revealed epileptic bilateral fronto-temporal encephalopathy, so that lacosamide was added. Levetiracetam daily dose was also adjusted, based on results of plasma concentration assays. Onset of fever was treated with antipyretic drugs and non-pharmacological interventions. No pathogens were grown on CSF and blood cultures.

To assess neurological impairment, pharmacological sedation was progressively de-escalated 48 hours after ICU admission; only on day 6, however, after adequate pharmacological control of epilepsy, improvement of her neurological status was observed, with cranial reflexes evocable, and simple order execution; extubation was performed and conventional oxygen therapy started.

Further neurological and psychiatric assessment revealed retrograde amnesia (past 2 years), mild apraxia and anisocoria. EEG controls evidenced persistent asynchronous epileptic abnormalities, associated with right temporal focuses. Rehabilitation with physiokinesis was started. On day 8, the patient was discharged in ameliorated general and neurological conditions, with a diagnosis of chemical meningo-encephalitis. Before discharge, she reported receiving a first adenoviral vaccine dose for SARS-CoV-2 a few hours in advance of receiving her spinal injection. Neither her blood parameters, nor EEGs and neuroimaging, however, revealed any alteration possibly suggestive of a post vaccine adverse reaction. At follow-up visits, the patient reported rescuing her ordinary work activities after 60 days, with no retrograde amnesia left.

## Discussion

Rare and serious complications of undesired intrathecal injections have been reported in literature, such as cauda equina syndrome and paraplegia [2]. Prognosis of UII may vary, depending on drug and dosage, since UII may cause a wide range of effects, from no complication, up to permanent neurological damage and death [2].

Although the absolute frequency of these complications may be rare, risks of UII should be always taken into consideration [3]. Staal *et al.* suggested that the effectiveness of injection therapy for low back pain is well proved. Due to heterogeneity of target tissues, pharmacological agents and dosages used, however, specific subgroups of patients may well exhibit abnormal reactions to intrathecal injection therapy. As a consequence, only one guideline, from Belgium, recommends injection therapy for the management of low back pain, whereas other guidelines from the USA, Europe, the UK, and Italy do not [4]. Instead, they recommend education plans about low back pain, exercises supervised by a medical specialist or therapist, NSAIDs, opioid analgesics, multidisciplinary rehabilitation, and behavioral therapy, to prevent the possible consequences that local anesthetics inadvertently injected in the subarachnoid space may cause [2, 4–6].

Thiocolchicoside is a semisynthetic derivative of colchicoside that exhibits selective affinity for both gamma-aminobutyric acid and glycinergic receptors. Its use as a muscle relaxant was authorized by national procedures in several EU Member States for the treatment of painful muscular disorders, orally or by intramuscular injection [12]. Side effects of thiocolchicoside include nausea, allergy, and vasovagal reactions [11]. Liver injury has also been rarely described [11]. The European Medicine Agency also indicated that thiocolchicoside may reduce fertility in men and, if administered during pregnancy, harm fetal embriogenesis. Based on *in vitro* studies, long-term medical exposure to thiocolchicoside might increase the risk of cancer, although direct evidence is currently lacking [12]. Most of all, in terms of relevance for the purpose of the present case, thiocolchicoside may have strong epileptogenic activity both in humans and in experimental animals, and it should be avoided in patients with possible disruptions of blood–brain barrier or a history of epilepsy. The relative impermeability of the blood–brain barrier (BBB) to therapeutic intramuscular doses of thiocolchicoside may explain why its epileptogenic activity has not been long pointed out, in spite of its extensive use in clinical practice for more than 30 years [13]. Acetyl-l-carnitine is a naturally occurring substance that promotes peripheral nerve regeneration with analgesic effects in patients with peripheral neuropathies of diabetic, HIV-related, or chemotherapeutic origin [14]. Is local or intrathecal use, however, has never been associated with chemical meningoencephalitis.

To our knowledge, this is the first reported case of inadvertent IT thiocolchicoside and l-acetylcarnitine injection in an adult patient, as well as the first in the neurosurgical literature [2]. With reasonable certainty, on the basis of the quite different neurotoxicity profiles of the two drugs, thiocolchicoside may well be reckoned to be the culprit in our patient. While she had a prompt recovery during her ICU stay by day 8, a major residual neurological deficit after 6 months of UII was represented by severe retrograde amnesia (RA). RA refers to loss of memory for information acquired before the onset of amnesia. This condition is commonly observed after medial temporal lobe or diencephalic insults. RA may affect recent and remote memories alike, and sometimes has been described as affecting both semantic memory and autobiographical, episodic memory. Post-encephalitic amnesic syndrome has been described in herpes virus encephalitis, at times with profound memory impairment; our patient suffered a well documented, short lasting episode of bilateral fronto-temporal epileptic encephalopathy, and might have undergone a similar pathogenetic damage as in viral encephalitis [8–10].

Management of chemical meningitis may include high dose of steroids, in association with other symptomatic treatments such as antiemetic drugs and analgesics [7]. Cerebrospinal fluid lavage may be taken into consideration, being indicated for UII of most drugs, including large dose of local anesthetics [2, 7]. In our case, cerebrospinal fluid lavage was not performed, because of the relatively rapid improvement of our patient’s global neural condition.

## Conclusions

We report on a well-characterized case of UII of thiocolchicoside, causing an abrupt onset, rapidly recovering chemical meningoencephalitis, complicated by early generalized seizures and causing long term, likely irreversible deep anterograde amnesia. Our experience suggests that injection therapy for low back pain should be administered only in adequate settings, where immediate rescue of possible complications may be possible.

## Data Availability

Data are available from the corresponding author upon reasonable request.
